# Supplementary material for: Plasma Lipid Profiles Change with Increasing Numbers of Mild Traumatic Brain Injuries in Rats
Source: Metabolites. 2022 Apr 2;12(4):322. doi: 10.3390/metabo12040322 (PMC9025508; doi:10.3390/metabo12040322)
Supplement: Supplementary file 1 [file metabolites-12-00322-s001.zip › metabolites-1603962-supplementary.pdf]

Supplement: Plasma Lipid Profiles Change with Increasing Numbers of Mild Traumatic Brain Injuries in Rats

Chidozie C. Anyaegbu [1,2,†], Harrison Szemray [3,†], Sarah C. Hellewell [1,2,*,†], Nathan G. Lawler [3,4], Kerry Leggett [2], Carole Bartlett [1], Brittney Lins [1,2], Terence McGonigle [1], Melissa Papini [1], Ryan S. Anderton [2,5], Luke Whiley [2,4,5,*] and Melinda Fitzgerald [1,2,*]

1 Curtin Health Innovation Research Institute, Curtin University, Bentley, WA 6102, Australia;
chidozie.anyaegbu@curtin.edu.au (C.C.A.); carole.bartlett@curtin.edu.au (C.B.);
brittney.lins@curtin.edu.au (B.L.); terence.mcgonigle@curtin.edu.au (T.M.);
melissa.papini@curtin.edu.au (M.P.)

2 Perron Institute for Neurological and Translational Science, Ralph and Patricia Sarich Neuroscience
Research Institute Building, Nedlands, WA 6009, Australia; kerry.leggett@uwa.edu.au (K.L.);
ryan.anderton@nd.edu.au (R.S.A.)

3 School of Health Sciences and Institute for Health Research, The University of Notre Dame Australia,
Fremantle, WA 6160, Australia; 34255866@student.murdoch.edu.au (H.S.);
nathan.lawler@murdoch.edu.au (N.G.L.)

4 Australian National Phenome Centre, Health Futures Institute, Murdoch University, Murdoch, WA 6150, Australia

5 Centre for Computational and Systems Medicine, Health Futures Institute, Murdoch University,
Murdoch, WA 6150, Australia

* Correspondence: sarah.hellewell@curtin.edu.au (S.C.H.); luke.whiley@murdoch.edu.au (L.W.);
melinda.fitzgerald@curtin.edu.au (M.F.)

† These authors contributed equally to this work.

Supplementary Information

Methods

Liquid chromatography-mass spectrometry

Lipidomic analysis of the plasma sample extracts was performed using a targeted tandem mass spectrometry (MS/MS) approach using predefined multiple reaction monitoring (MRM) transitions (Sciex sMRM Pro Builder, Framingham, MA, USA) and in-house defined chromatographic retention time windows.

The analytical system consisted of a Sciex ExionLC ™ coupled to a Triple Quadrupole Linear Ion Trap (QTRAP 6500+) (SCIEX, Concord, ON, Canada).

Chromatographic separation was achieved using a Waters Acquity BEH C18 Column (1.7 µm, 100 x 2.1 mm particle size; Waters Corp.), maintained at 60°C. The

gradient consisted of 20/30/50, Isopropanol/Acetonitrile/H_2O + 10 mM Ammonium Acetate (A) and 90/9/1, Isopropanol/Acetonitrile/ H_2O + 10 mM Ammonium Acetate (B). The solvent was delivered at a flow rate of 0.4mL/min. The gradient consisted of 10% B at 0 minutes, 1 to 45% B in 2.7 minutes, 45 to 53% B in 0.1 minutes, 53 to 60% B in 5.2 minutes, 60 to 80% in 0.1 minutes, maintained at 80% B for 3.4 minutes, 80 to 100% B in 0.5 minutes, maintained at 100% B for 1 minute, and then equilibration at initial conditions for 1.99 minutes.

A Sciex QTRAP 6500+ was operated with electrospray ionisation using polarity switching. The following mass spectrometer settings were used; capillary voltage, 5500 V (positive ion mode) and -4500 V (negative ion mode); temperature, 300 °C; curtain gas, 20 psi; ion source gas 1, 40 psi; ion source gas 2, 60 psi. Time-scheduled multiple reaction monitoring (MRM) was used for data acquisition. Data was acquired using Analyst®1.7.1 (QTRAP 6500+ LC-MS/MS system) (SCIEX, Concord, ON, Canada). Specific MS settings, MRM transitions and chromatographic retention times for the lipids of interest reported in the results section and Tables S2-S4 are included in Table S5.

Results

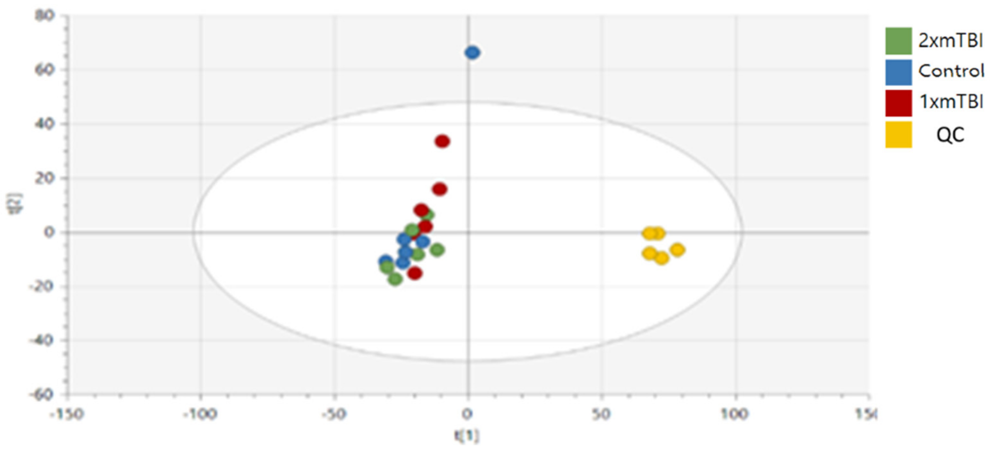

Figure S1. Unsupervised Principal Component Analysis (PCA) with 19 samples (6 from sham controls, 6 from rats with 1xmTBI, and 7 from rats with 2xmTBI). The yellow circles represent repeat extractions of a quality control (QC) pooled sample (n = 5). The QC used was human plasma and therefore clustering separate to the rat samples. Clustering of the QC indicated consistent analysis throughout the run. Two samples (overlayed in the PCA) were identified as outliers and removed from subsequent statistical analysis.

Table S1. Variable Influence on Projection (VIP) values generated for the OPLS-DA model presented in manuscript Figure 1A (control vs 1x mTBI). The table includes the top 20 lipid features (ordered by VIP value). Jack-knife standard error of the VIP values are also included in the table (VIP cvSE).

Lipid	VIP	VIP cvSE
HCER(D18:0/26:0)	2.26	1.65
PS(14:0/18:2)	2.20	0.83
PI(16:0/18:3)	2.00	1.77
PI(16:0/20:2)	1.94	1.73
CE(22:4)	1.92	1.48
FFA(22:5)	1.90	1.83
PC(18:0/22:4)	1.86	1.78
FFA(20:3)	1.83	1.75
DAG(18:2/22:5)	1.8	2.00
SM(24:0)	1.77	1.32
PG(18:0/20:0)	1.77	0.62
PC(18:0/18:1)	1.77	1.65
CE(14:0)	1.76	1.08
PE(16:0/20:3)	1.75	1.89
PC(18:1/22:4)	1.75	0.87
PC(18:2/22:5)	1.74	1.52
PC(14:0/20:4)	1.69	1.56
PC(14:0/18:2)	1.69	1.49
PG(20:0/20:4)	1.68	0.98
TAG(45:0/FA16:0)	1.68	1.27

Table S2. Variable Influence on Projection (VIP) values generated for the OPLS-DA model presented in manuscript Figure 1B (control vs 2x mTBI). The table includes the top 20 lipid features (ordered by VIP value). Jack-knife standard error of the VIP values are also included in the table (VIP cvSE).

**Lipid**	**VIP**	**VIP cvSE**
PS(14:0/18:2)	2.06	0.85
LPI(18:2)	1.82	2.62
CE(14:0)	1.82	1.34
PI(16:0/20:2)	1.81	1.85
PI(16:0/18:2)	1.78	2.17
LPE(20:1)	1.74	2.88
PI(20:0/18:2)	1.66	0.84
PC(20:0/22:6)	1.62	2.80
FFA(16:2)	1.62	1.96
HCER(d18:0/26:0)	1.61	0.76
PG(20:0/20:4)	1.61	3.20
PI(18:2/20:1)	1.58	1.23
PI(18:1/18:2)	1.57	1.11
PC(18:2/22:5)	1.56	1.85
PE(O-18:0/22:5)	1.55	1.67
LPE(20:4)	1.55	1.33
LPG(18:2)	1.53	1.27
TAG(42:0/FA16:0)	1.51	2.02
TAG(53:4/FA20:4)	1.50	1.53
CE(20:0)	1.48	2.09

Table S3. Variable Influence on Projection (VIP) values generated for the OPLS-DA model presented in manuscript Figure 1C (1x mTBI vs 2x mTBI). The table includes the top 20 lipid features (ordered by VIP value). Jack-knife standard error of the VIP values are also included in the table (VIP cvSE).

**Lipid**	**VIP**	**VIP cvSE**
LPE(22:6)	1.85	0.74
LPE(20:4)	1.76	0.64
DCER(24:1)	1.67	2.37
LPE(18:2)	1.66	0.76
LPE(18:3)	1.63	1.05
TAG(57:3/FA18:2)	1.62	0.58
TAG(55:1/FA16:0)	1.61	0.85
TAG(57:2/FA18:1)	1.60	0.54
LPE(18:1)	1.59	0.82
LPE(22:5)	1.58	0.68
TAG(56:2/FA16:0)	1.57	0.80
TAG(55:2/FA18:2)	1.57	0.99
TAG(56:1/FA16:0)	1.56	0.99
LPE(20:1)	1.55	0.83
PE(P-16:0/20:3)	1.55	1.84
TAG(54:1/FA16:0)	1.54	0.64
TAG(55:1/FA18:1)	1.54	0.78
PE(P-18:0/20:4)	1.53	2.58
PI(16:0/18:3)	1.52	0.87
TAG(58:2/FA18:1)	1.51	0.72

Table S4. Mass Spectrometry Multiple Reaction Monitoring (MRM) transitions and chromatographic retention times for the lipids of interest reported in the results section of the manuscript and Supplementary Tables S2–S4. Collision Energy (CE), Declustering Potential (DP), Entrance Potential (EP), Collision Cell Exit Potential (CXP), Internal Standard (ISTD).

Subclass	Lipid	RT (min)	Q1 Mass (Da)	Q3 mass (Da)	Adduct	CE	DP	EP	CXP	Internal Standard (ISTD)
CE	CE(14:0)	11.63	614.6	369.4	[M+H]+	25	60	10	15	CE(16:0)-d7
CE	CE(20:0)	13.04	698.7	369.4	[M+H]+	25	60	10	15	CE(20:3)-d7
CE	CE(22:4)	11.95	718.6	369.4	[M+H]+	25	60	10	15	CE(22:6)-d7
DAG	DAG(18:2/22:5)	8.81	684.6	337.3	[M+NH4]+	26	60	10	15	DAG(16:0/22:6)-d9
DCER	DCER(24:1)	9.74	650.9	266.4	[M+H]+	43	60	10	15	DCER(16:0)-d9
FFA	FFA(16:2)	2.24	251.1	251.1	[M-H]-	-10	-80	-15	-10	FFA(16:0)-d9
FFA	FFA(18:3)	2.50	277.2	277.2	[M-H]-	-10	-80	-15	-10	FFA(16:0)-d9
FFA	FFA(20:3)	3.27	305.2	305.2	[M-H]-	-10	-80	-15	-10	FFA(16:0)-d9
FFA	FFA(22:5)	3.07	329.2	329.2	[M-H]-	-10	-80	-15	-10	FFA(16:0)-d9
HCER	HCER(22:0)	9.52	784.9	264.4	[M+H]+	43	60	10	15	HCER(16:0)-d9
HCER	HCER(d18:0/26:0)	9.84	842.9	266.4	[M+H]+	43	60	10	15	HCER(16:0)-d9
LPE	LPE(18:1)	3.16	478.3	281.2	[M-H]-	-40	-80	-15	-10	LPE(18:1)-d7
LPE	LPE(18:2)	2.64	476.3	279.2	[M-H]-	-40	-80	-15	-10	LPE(18:1)-d7
LPE	LPE(18:3)	2.21	474.3	277.2	[M-H]-	-40	-80	-15	-10	LPE(18:1)-d7
LPE	LPE(20:1)	3.79	506.3	309.3	[M-H]-	-40	-80	-15	-10	LPE(18:1)-d7
LPE	LPE(20:4)	2.58	500.3	303.2	[M-H]-	-40	-80	-15	-10	LPE(18:1)-d7
LPE	LPE(22:5)	2.73	526.3	329.2	[M-H]-	-40	-80	-15	-10	LPE(18:1)-d7
LPE	LPE(22:6)	2.50	524.3	327.2	[M-H]-	-40	-80	-15	-10	LPE(18:1)-d7
LPG	LPG(18:2)	2.22	507.3	279.2	[M-H]-	-50	-80	-15	-10	LPG(17:1)-d7
LPI	LPI(18:2)	2.07	595.3	279.2	[M-H]-	-50	-80	-15	-10	LPG(17:1)-d7
PC	PC(14:0/18:2)	6.03	788.5	279.2	[M+OAc]-	-50	-80	-15	-10	PC(15:0/18:1)-d7
PC	PC(14:0/20:4)	5.93	812.5	303.2	[M+OAc]-	-50	-80	-15	-10	PC(15:0/18:1)-d7
PC	PC(18:0/18:1)	9.15	846.6	281.2	[M+OAc]-	-50	-80	-15	-10	PC(16:0/18:1)-d9
PC	PC(18:0/22:4)	9.07	896.6	331.3	[M+OAc]-	-50	-80	-15	-10	PC(16:0/22:4)-d9
PC	PC(18:1/22:4)	7.88	894.6	331.3	[M+OAc]-	-50	-80	-15	-10	PC(16:0/22:4)-d9
PC	PC(18:2/22:5)	6.34	890.6	329.2	[M+OAc]-	-50	-80	-15	-10	PC(16:0/22:5)-d9
PC	PC(20:0/22:4)	9.39	924.7	331.3	[M+OAc]-	-50	-80	-15	-10	PC(16:0/22:4)-d9
PC	PC(20:0/22:6)	9.09	920.6	327.2	[M+OAc]-	-50	-80	-15	-10	PC(16:0/22:6)-d9
PE	PE(16:0/20:3)	7.64	740.5	305.2	[M-H]-	-50	-80	-15	-10	PE(15:0/18:1)-d7
PE	PE(O-18:0/22:5)	9.16	778.6	329.2	[M-H]-	-50	-80	-15	-10	PE(18:0/22:5)-d5
PE	PE(P-16:0/20:3)	8.26	724.5	305.2	[M-H]-	-50	-80	-15	-10	PE(18:0/20:3)-d5
PE	PE(P-18:0/20:1)	9.62	756.6	309.3	[M-H]-	-50	-80	-15	-10	PE(18:0/20:3)-d5
PE	PE(P-18:0/20:4)	9.06	750.5	303.2	[M-H]-	-50	-80	-15	-10	PE(18:0/20:4)-d5
PE	PE(P-18:1/20:4)	7.86	748.5	303.2	[M-H]-	-50	-80	-15	-10	PE(18:0/20:4)-d5
PG	PG(18:0/20:0)	8.12	805.6	283.3	[M-H]-	-50	-80	-15	-10	PG(15:0/18:1)-d7

PG	PG(20:0/20:4)	7.44	825.6	303.2	[M-H]-	-50	-80	-15	-10	PG(15:0/18:1)-d7
PI	PI(16:0/18:2)	5.43	833.5	279.2	[M-H]-	-60	-80	-15	-10	PG(15:0/18:1)-d7
PI	PI(16:0/18:3)	5.07	831.5	277.2	[M-H]-	-60	-80	-15	-10	PG(15:0/18:1)-d7
PI	PI(16:0/20:2)	6.18	861.6	307.3	[M-H]-	-60	-80	-15	-10	PG(15:0/18:1)-d7
PI	PI(18:1/18:1)	6.13	861.6	281.2	[M-H]-	-60	-80	-15	-10	PG(15:0/18:1)-d7
PI	PI(18:1/18:2)	5.52	859.5	279.2	[M-H]-	-60	-80	-15	-10	PG(15:0/18:1)-d7
PI	PI(18:2/20:1)	6.24	887.6	309.3	[M-H]-	-60	-80	-15	-10	PG(15:0/18:1)-d7
PI	PI(20:0/18:2)	5.95	889.6	279.2	[M-H]-	-60	-80	-15	-10	PG(15:0/18:1)-d7
PS	PS(14:0/18:2)	6.82	730.5	279.2	[M-H]-	-50	-80	-15	-10	PG(15:0/18:1)-d7
SM	SM(24:0)	9.69	815.7	184.1	[M+H]+	43	60	10	15	SM(24:0)-d7
TAG	TAG(42:0/FA16:0)	10.82	740.7	467.4	[M+NH4]+	38	60	10	15	TAG(50:1/FA16:0)-d9
TAG	TAG(45:0/FA16:0)	11.51	782.7	509.5	[M+NH4]+	38	60	10	15	TAG(50:1/FA16:0)-d9
TAG	TAG(53:4/FA20:4)	11.49	886.8	565.5	[M+NH4]+	38	60	10	15	TAG(54:4/FA20:3)-d9
TAG	TAG(54:1/FA16:0)	12.87	906.8	633.5	[M+NH4]+	38	60	10	15	TAG(50:1/FA16:0)-d9
TAG	TAG(55:1/FA16:0)	13.06	920.9	647.6	[M+NH4]+	38	60	10	15	TAG(50:1/FA16:0)-d9
TAG	TAG(55:1/FA18:1)	12.96	920.9	621.6	[M+NH4]+	38	60	10	15	TAG(52:2/FA18:1)-d9
TAG	TAG(55:2/FA18:2)	12.96	918.8	621.5	[M+NH4]+	38	60	10	15	TAG(52:3/FA18:2)-d9
TAG	TAG(56:1/FA16:0)	13.12	934.9	661.6	[M+NH4]+	38	60	10	15	TAG(50:1/FA16:0)-d9
TAG	TAG(56:2/FA16:0)	13.01	932.9	659.6	[M+NH4]+	38	60	10	15	TAG(50:1/FA16:0)-d9
TAG	TAG(57:2/FA18:1)	13.06	946.9	647.6	[M+NH4]+	38	60	10	15	TAG(52:2/FA18:1)-d9
TAG	TAG(57:3/FA18:2)	12.95	944.9	647.6	[M+NH4]+	38	60	10	15	TAG(52:3/FA18:2)-d9
TAG	TAG(58:2/FA18:1)	13.12	960.9	661.6	[M+NH4]+	38	60	10	15	TAG(52:2/FA18:1)-d9
**CE**	**CE(16:0)-d7**	**12.26**	**649.6**	**376.5**	**[M+H]+**	**25**	**60**	**10**	**15**	**ISTD**
**CE**	**CE(20:3)-d7**	**11.81**	**699.6**	**376.5**	**[M+H]+**	**25**	**60**	**10**	**15**	**ISTD**
**CE**	**CE(22:6)-d7**	**11.21**	**721.6**	**376.5**	**[M+H]+**	**25**	**60**	**10**	**15**	**ISTD**
**DAG**	**DAG(16:0/22:6)-d9**	**8.76**	**667.5**	**322.2**	**[M+NH4]+**	**26**	**60**	**10**	**15**	**ISTD**
**DCER**	**DCER(16:0)-d9**	**8.38**	**549.6**	**266.4**	**[M+H]+**	**43**	**60**	**10**	**15**	**ISTD**
**FFA**	**FFA(16:0)-d9**	**3.35**	**264.1**	**264.1**	**[M-H]-**	**-10**	**-80**	**-15**	**-10**	**ISTD**
**HCER**	**HCER(16:0)-d9**	**6.97**	**709.7**	**264.4**	**[M+H]+**	**43**	**60**	**10**	**15**	**ISTD**
**LPE**	**LPE(18:1)-d7**	**3.14**	**485.3**	**288.3**	**[M-H]-**	**-40**	**-80**	**-15**	**-10**	**ISTD**
**LPG**	**LPG(17:1)-d7**	**2.33**	**495.3**	**267.2**	**[M-H]-**	**-50**	**-80**	**-15**	**-10**	**ISTD**
**PC**	**PC(15:0/18:1)-d7**	**7.29**	**811.6**	**288.3**	**[M+OAc]-**	**-50**	**-80**	**-15**	**-10**	**ISTD**
**PC**	**PC(16:0/18:1)-d9**	**7.88**	**827.6**	**281.2**	**[M+OAc]-**	**-50**	**-80**	**-15**	**-10**	**ISTD**
**PE**	**PC(16:0/22:4)-d9**	**7.71**	**877.6**	**331.2**	**[M+OAc]-**	**-50**	**-80**	**-15**	**-10**	**ISTD**
**PC**	**PC(16:0/22:5)-d9**	**6.95**	**875.6**	**329.2**	**[M+OAc]-**	**-50**	**-80**	**-15**	**-10**	**ISTD**
**PC**	**PC(16:0/22:6)-d9**	**6.64**	**873.6**	**327.2**	**[M+OAc]-**	**-50**	**-80**	**-15**	**-10**	**ISTD**
**PE**	**PE(15:0/18:1)-d7**	**7.56**	**709.6**	**288.3**	**[M-H]-**	**-50**	**-80**	**-15**	**-10**	**ISTD**
**PE**	**PE(18:0/20:3)-d5**	**8.91**	**773.6**	**305.2**	**[M-H]-**	**-50**	**-80**	**-15**	**-10**	**ISTD**
**PE**	**PE(18:0/20:4)-d5**	**8.34**	**771.5**	**303.2**	**[M-H]-**	**-50**	**-80**	**-15**	**-10**	**ISTD**
**PE**	**PE(18:0/22:5)-d5**	**8.45**	**797.6**	**329.2**	**[M-H]-**	**-50**	**-80**	**-15**	**-10**	**ISTD**
**PG**	**PG(15:0/18:1)-d7**	**5.64**	**740.6**	**288.3**	**[M-H]-**	**-50**	**-80**	**-15**	**-10**	**ISTD**
**SM**	**SM(24:0)-d7**	**9.68**	**822.7**	**184.2**	**[M+H]+**	**43**	**60**	**10**	**15**	**ISTD**

**TAG**	**TAG(50:1/FA16:0)-d9**	**12.37**	**859.8**	**586.6**	**[M+NH4]+**	**38**	**60**	**10**	**15**	**ISTD**
**TAG**	**TAG(52:2/FA18:1)-d9**	**12.39**	**885.8**	**586.6**	**[M+NH4]+**	**38**	**60**	**10**	**15**	**ISTD**
**TAG**	**TAG(52:3/FA18:2)-d9**	**11.87**	**883.8**	**586.6**	**[M+NH4]+**	**38**	**60**	**10**	**15**	**ISTD**
**TAG**	**TAG(54:4/FA20:3)-d9**	**12.00**	**909.8**	**586.6**	**[M+NH4]+**	**38**	**60**	**10**	**15**	**ISTD**

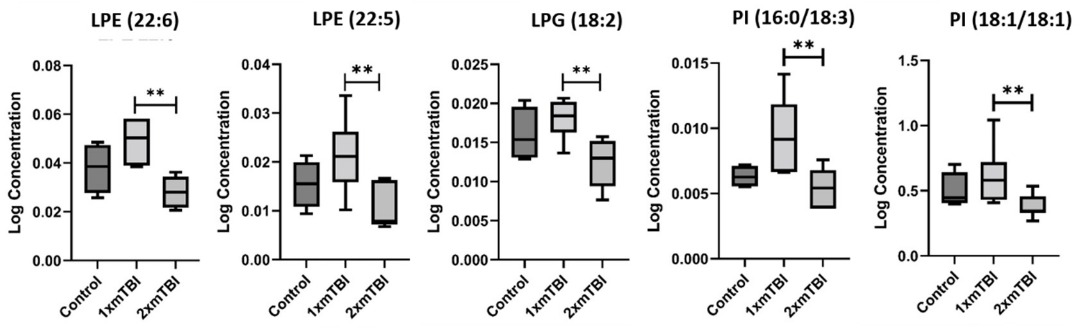

Figure S2. Lipids that decrease between one mTBI and two mTBIs. Boxplots represent the minimum, first quartile, median, third quartile, and maximum, for the control, 1xmTBI and 2xmTBI groups. Significant differences between groups are indicated above the boxplots by ** (p<0.01).

Table S5. A non-exhaustive list of lipid class findings in clinical or experimental studies in mild and moderate-severe TBI.

	Altered in plasma or serum after Human TBI	Altered in plasma or serum after experimental TBI	Altered in brain tissue after experimental TBI
**CE**	-	↑ 11 days in single rotational mTBI<br>↑ 11 days in rotational rmTBI	↑ 3 & 7 after moderate-severe controlled cortical impact TBI [32]
**FFA**	-	↑ 3 & 7 days after moderate-severe controlled cortical impact TBI [37]<br>↑ 11 days in single rotational mTBI	-
**HCER**	-	↓ 11 days in single rotational mTBI<br>↓ 11 days in rotational rmTBI – HCER(d18:0/26:0)<br>↑ 11 days in rotational rmTBI - HCER(22:0)	-
**LPE**	↓ chronically after mTBI [16]	↓ 11 days in rotational rmTBI<br>↓ over 12 months in closed head mTBI [10]	↑ acutely and chronically in rmTBI [17]
**PC**	↓ 55 hours after mTBI [15]<br>↓ chronically after mTBI [16]	↓ over 24 hours in controlled cortical impact mTBI & rmTBI [42]<br>↓ 3 & 7 days after moderate-severe controlled cortical impact TBI [37]<br>↓ 11 days in rotational rmTBI<br>↓ 3 months in moderate-severe controlled cortical impact [41]<br>↓ over 12 months in closed head mTBI [10]	↑ 3 days in lesion and perilesional tissue in moderate-severe controlled cortical [40]<br>↑ 24 hours to 12 months in rmTBI [17]<br>↓ 3 months in moderate-severe controlled cortical impact [41]
**PE**	↓ chronically after mTBI [16]	↓ over 24 hours in controlled cortical impact mTBI & rmTBI [42]<br>↓ 3 & 7 days after moderate-severe controlled cortical impact TBI [37]<br>↑ 11 days in rotational rmTBI<br>↓ 3 months in moderate-severe controlled cortical impact [41]<br>↓ over 12 months in closed head mTBI [10]	↑ 3 days in perilesional tissue in moderate-severe controlled cortical [40]<br>↑ 24 hours to 12 in rmTBI [17]<br>↑ 24 hours to 12 months in rmTBI [43]<br>↓ 3 months in moderate-severe controlled cortical impact [41]
**PI**	↓ chronically after mTBI [16]	↑ 11 days in rotational single mTBI<br>↓ 11 days in rotational rmTBI<br>↓ over 12 months in closed head mTBI [10]	-
**PS**	-	↓ over 24 hours in controlled cortical impact mTBI & rmTBI [42]	-

		↓ 11 days in rotational single mTBI<br>↓ 11 days in rotational rmTBI<br>↑ 3 & 7 days in serum after moderate-severe controlled cortical impact TBI [37]	

Red arrows and text indicate new findings in this study. ↑ lipid class increased in study relative to uninjured control; ↓ lipid class decreased in study relative to uninjured control; *CE, cholesterol ester; FFA, free fatty acid; HCER, hexosylceramide; LPE, lysophosphatidylethanolamine; mTBI, mild traumatic brain injury; PC, phosphocholine; PE, phosphoethanolamine; PI, phosphoinositol; PS, phosphoserine; rmTBI, repetitive mild traumatic brain injury. Note: new findings for HCER listed at species level due to opposing findings within class.*
